# Relationship between Bone Stability and Egg Production in Genetically Divergent Chicken Layer Lines

**DOI:** 10.3390/ani10050850

**Published:** 2020-05-14

**Authors:** Simon Jansen, Ulrich Baulain, Christin Habig, Annett Weigend, Ingrid Halle, Armin Manfred Scholz, Henner Simianer, Ahmad Reza Sharifi, Steffen Weigend

**Affiliations:** 1 Institute of Farm Animal Genetics, Friedrich-Loeffler-Institut, 31535 Neustadt, Germany; ulrich.baulain@fli.de (U.B.); christin.habig@fli.de (C.H.); annett.weigend@fli.de (A.W.); steffen.weigend@fli.de (S.W.); 2 Institute of Animal Nutrition, Friedrich-Loeffler-Institut, 38116 Braunschweig, Germany; ingrid.halle@fli.de; 3 Livestock Center of the Faculty of Veterinary Medicine, Ludwig-Maximilians-University Munich, 85764 Oberschleissheim, Germany; armin.scholz@lvg.vetmed.uni-muenchen.de; 4 Animal Breeding and Genetics Group, Department of Animal Sciences, University of Göttingen, 37075 Göttingen, Germany; 5 Center for Integrated Breeding Research, University of Göttingen, 37075 Göttingen, Germany

**Keywords:** animal welfare, bone mineral density, bone breaking strength, fractures, laying hens, laying performance, osteoporosis, phylogeny

## Abstract

**Simple Summary:**

Brittle or fractured bones due to continuous demineralisation cause major welfare and economic problems in laying hens. Bone weakness in laying hens is frequently attributed to long-term selection for increased egg production, but this is controversially discussed in the scientific literature. We aimed at characterizing factors influencing the bone breaking strength of laying hens, focusing mainly on the effect of eggshell production. By examining four different chicken layer lines separately, a genetically diverse spectrum of laying hen origins was included in our study. It was shown that bone strength is primarily influenced by bone mineral density. A strong association between bone strength and eggshell production was not observed within each of the lines studied. This applied to all layer lines. Our results suggest that a high egg number does not generally impair bone stability within layer lines. Findings from this study contribute to the discussion on the improvement of bone stability in poultry breeding programs and thus lead to increased animal welfare in egg production.

**Abstract:**

Impaired animal welfare due to skeletal disorders is likely one of the greatest issues currently facing the egg production industry. Reduced bone stability in laying hens is frequently attributed to long-term selection for increased egg production. The present study sought to analyse the relationship between bone stability traits and egg production. The study comprised four purebred layer lines, differing in their phylogenetic origin and performance level, providing extended insight into the phenotypic variability in bone characteristics in laying hens. Data collection included basic production parameters, bone morphometry, bone mineral density (BMD) and bone breaking strength (BBS) of the tibiotarsus and humerus. Using a multifactorial model and regression analyses, BMD proved to be of outstanding importance for bone stability. Only for the tibiotarsus were morphometric parameters and the bone weight associated with BBS. Within the chicken lines, no effect of total eggshell production on BBS or BMD could be detected, suggesting that a high egg yield itself is not necessarily a risk for poor bone health. Considering the complexity of osteoporosis, the estimated genetic parameters confirmed the importance of genetics in addressing the challenge of improving bone strength in layers.

## 1. Introduction

Although the consideration of functional traits in selection programs to improve animal health has become increasingly important in recent years, the number of saleable eggs and extended persistency of laying are still the main goals in the breeding of laying hens [1]. Since up to three grams of calcium per egg are required for eggshell formation [2], laying more than 300 eggs in 12 months in highly selected commercial hens is an immense challenge for the calcium homeostasis, and as part of it, the skeletal system of the bird. During eggshell calcification, laying hens cover, partially, the temporarily high demand of calcium with increased mobilisation from the bones [3]. In avian species, medullary bone serves as a labile calcium source and its formation increases with the onset of sexual maturity [4,5]. However, this is accompanied by a decrease of cancellous bone volume under the influence of oestrogen [6,7,8]. Continuous demineralisation leads to osteoporosis, a pathological condition of progressive loss of structural bone tissue, resulting in brittle and fragile bones being susceptible to fractures [3,9].

High incidences of birds suffering from osteoporotic or fractured bones have been reported [10,11,12]. Riber et al. [13] concluded that hens suffering from bone fractures show marked atypical behavioural differences compared to those with healthy bones, suggesting that osteoporosis has serious animal welfare implications. Nasr et al. [14] proved that hens with keel bone fractures do experience pain. Bone weakness can also be a cause of mortality, as shown in a study by McCoy et al. [15], in which it accounted for up to 35% of the deaths. Fracture-associated decline in performance adds an economic dimension to the implications of skeletal disorders [8,16,17].

In addition to the important factors of nutrition and husbandry, genetics are considered a decisive factor for bone health [3,18,19]. Skeletal problems in layers are frequently attributed to selection for increased egg production, suggesting a negative association between laying performance and bone stability [8,20,21,22,23,24]. As bone quality traits are supposed to be highly polygenic [18], genetic correlations might lead to an accompaniment of selection for high laying performance by undesirable “co-selectional” side effects [25,26,27]. In the case of calcium homeostasis, this may have resulted in a prioritization of calcium resources in favour of reproduction and to the disadvantage of bone health [28,29].

Targeted genetic selection certainly makes the main contribution to changes in performance potential and may be associated with undesirable associated effects on bone stability. However, differences may also be due to the phylogenetic origin of these lines, whose distinct breeding history may have influenced the genetic characteristics before selection for high performance began. Since white and brown-egg laying chicken lines evolved separately after domestication from red jungle fowl several thousands of years ago and underwent genomic changes [30,31], phylogenetic origin has potential implications for bone characteristic differences [32]. Therefore, in addition to the comparison of genetic lines, it is necessary to assess the association within the genetic lines.

This study is part of a multidisciplinary collaboration initiated at the Friedrich-Loeffler-Institut to investigate the effects of selection on performance efficiency in terms of adaptability to changing environmental conditions in laying hens. The animal model used comprised four chicken lines, two white and two brown-egg layers, which are phylogenetically distant and evolved independently during breed history [31]. Within each phylogenetic group, the two lines differed in performance level, since one of them originated from a contemporary commercial egg layer breeding line (“high performing”), whereas its counterpart was based on a conservation flock without any selection for many generations (“moderate performing”) [33]. Within the framework of these research activities, the phenotypic data on bone stability and egg production were used for genetic analyses in the present study, which were collected from laying hens with complete pedigree in two consecutive generations of four chicken-layer lines. The animals were supplied with different amounts of vitamin D3 (cholecalciferol). The dietary vitamin D3 content was varied in tests, since a relationship between bone stability and vitamin D3 was assumed [4,9]. We aimed at analysing the relationship between bone stability traits and egg production within the genetically divergent layer lines used in this model. Based on the frequently stated negative association between bone stability and egg production, we hypothesized that the two high performing layer lines show deficits in bone stability compared to their moderately performing counterparts, and that within lines the level of eggshell production significantly contributes to the variation of bone breaking strength (BBS) in humerus and tibiotarsus.

## 2. Materials and Methods 

### 2.1. Ethical Note

The present experiment was performed in accordance with the German Animal Welfare Law and approved by the Lower Saxony State Office for Consumer Protection and Food Safety (LAVES) (33.19-42502-04-15/1988).

### 2.2. Animals and Housing

The study included four purebred chicken layer lines (*Gallus gallus domesticus*), which differed in respect to their phylogenetic origins and performance levels. Lines WLA and BLA originated from a commercial breeding program of the Lohmann Tierzucht GmbH (Cuxhaven, Germany) selected for high laying performance. These lines have been maintained in a sire rotation program since 2012 and achieve a laying rate of about 320 eggs per year. In contrast, lines L68 and R11 have been maintained as non-selected resource populations at the Institute of Farm Animal Genetics, Friedrich-Loeffler-Institut (Neustadt, Germany) for more than 25 generations, R11 even for more than 50 generations. Their laying performance is about 200 eggs per year [33]. In addition to performance differences, the animal model considered a phylogenetic component, since white-egg layer lines WLA and R11 (both originating from White Leghorn) are phylogenetically closely related, but distinct from brown-egg layer lines BLA and L68. BLA originates from Rhode Island Red, while L68 descends from New Hampshire, a breed that has been developed from Rhode Island Red chickens [31].

The experiment was conducted in two consecutive generations with 576 hens in total (72 hens per layer line and generation). All chicks of a respective replicate were hatched on the same day and were reared in floor pens of 24 m² until the 16th week of age. Information on the light program and the mean climatic conditions is given in the Supplementary Material (Table S1). Usual feeding stuff for chicks (until 6 weeks of age) and pullets (from 7 to 16 weeks of age), which had sufficient content of phosphorus, calcium and vitamin D3, was offered *ad libitum*. The nutrient compositions of these diets are listed in the Supplementary Material (Table S2).

After birds were transferred to the layer facility at the 16th week of age, they were kept in individual cages. The cage dimensions were 50 cm × 48 cm, which equals 2400 cm² of total floor space, and it was equipped with a plastic perch of 3 cm diameter. At the beginning of the 17th week of age, two customary wheat-soya-based diets for layers were fed *ad libitum*. The diets’ compositions and their nutrient contents are detailed in the Supplementary Material (Table S3). The two diets differed in content of vitamin D3: 300 IU, according to the recommendations of the German Society of Nutrition Physiology [34], or 3000 IU, displaying the maximum content according to the regulation (EC) number 1831/2003 of the European Parliament and of the Council [35]. It turned out, however, that no significant differences were found in terms of this difference in the vitamin D3 content for the traits studied (Table S4). It is possible that the difference between 300 and 3000 IU of vitamin D3 was not sufficient to elicit a response reflected in the observed characteristics, as both contents were within the range of what is considered to be adequate for chickens [36]. The results could indicate that laying hens may tolerate a wide range of dietary vitamin D3 supply. However, the present study cannot provide deeper insights into this. With regard to the genetic analysis presented here, data from both vitamin D3 groups were combined.

### 2.3. Experimental Procedure

**Figure 1:**
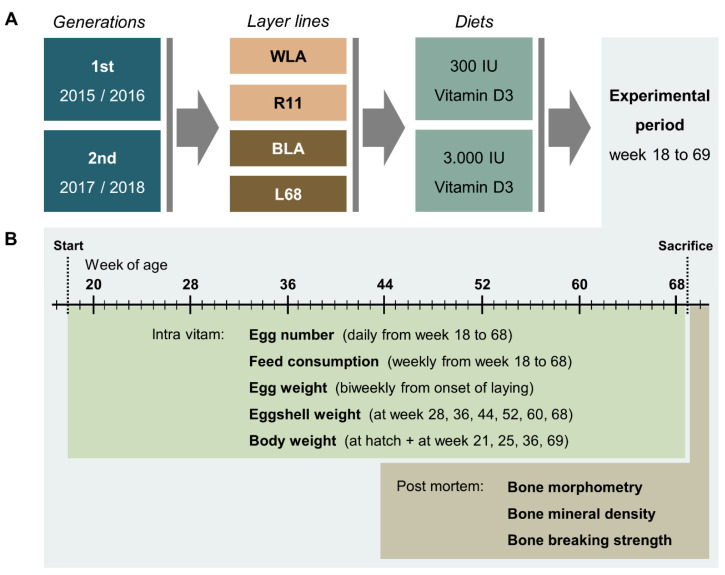
Schematic illustration of the experimental setup (**A**) and related data collection (**B**). In two consecutive generations, four chicken layer lines were allocated to a diet containing either 300 or 3000 IU of vitamin D3. During the experimental period, data on egg number, egg quality, feed consumption and body weight were collected as indicated. *Post mortem*, bone morphometry, bone mineral density and bone breaking strength were assessed.

The experimental setup, including data collection, is shown in Figure 1 and was identical in both generations. The experimental trial lasted 52 weeks from the 18th to the 69th week of age. The individual egg number was recorded daily during weeks 18 to 68; i.e., over 51 laying weeks. Egg weight data (g) were collected every two weeks over four consecutive days each, resulting in a mean egg weight value per individual (based on an average of 78.6 eggs per individual). Eggshell weight (g) was determined six times, at week 28, 36, 44, 52, 60 and 68, on four consecutive days each. For this, the eggs were emptied and shells were dried for 30 s in a microwave (800 watt). A digital table scale with a weighing accuracy of 0.01 g (Type 3709, Sartorius, Göttingen, Germany) was used for egg and eggshell weight determination. Eggshell proportion was calculated as the ratio between eggshell and egg weight. For the eggshell characteristics, mean values were calculated the same way as for the egg weight (based on an average of 18.6 eggs per individual). Total eggshell production was calculated by multiplying the mean eggshell weight and the total egg number. Feed consumption (g) was determined weekly on individual basis by back weighing the remaining feed using a table scale with a weighing accuracy of 20 g (Dexal 3, Epel Industrial, Sant Boi de Llobregat, Spain). Based on this, daily feed consumption (g) was calculated. Feed-to-egg conversion rate was calculated by dividing total feed consumption by the product of mean egg weight and total number of eggs. Feed-to-eggshell conversion rate was calculated analogously. Body weight (g) was measured at hatch and during the experimental period (at week 21, 25, 35 and 69) using a digital table scale (CPA 16001S, Sartorius, Göttingen, Germany) with a weighing accuracy of 0.1 g.

The hens were euthanized by carbon dioxide inhalation after 69 weeks of age. The left tibiotarsus and humerus were extracted and the adherent tissue removed. Bone weight (g), length (mm) and thickness (mm) were recorded, and bone mineral density (BMD) (g/cm²) was examined by dual energy X-ray absorptiometry (DXA) (GE Lunar *i*DXA scanner, GE Healthcare, Solingen, Germany). The bones were scanned and analysed by using the small animal mode within the enCore^®^ software version 17 (GE Healthcare, Solingen, Germany). A standardised rice pack positioned between dual energy X-ray source and bones served as the soft tissue standard. All bones were stored, transported and scanned under vacuum conditions in special plastic bags individually for each hen. Manually defined regions of interest helped to analyse the bones separately after the scan. Individual results were stored using the PDF and the DICOM file formats. BBS values (N) of the tibiotarsus and humerus were assessed at the mid-diaphyseal region via three-point bending test (Instron Materials Testing System, Instron Corporation, Canton, MA, USA). Thereby a 5 kN load cell was used and the span length was 40 mm (humerus) or 80 mm (tibiotarsus).

### 2.4. Statistical Analysis

As bones are supposed to differ noticeably depending on the reproductive status in female birds, exclusion of non-reproducing hens was required [18]. Individuals whose total egg numbers were outside the line specific threefold interquartile range (IQR) (<X_0.25_ − 3 × IQR; > X_0.75_ + 3 × IQR) and who did not lay an egg during the last three consecutive experimental weeks were considered as outliers. After filtering, a total number of 524 animals remained for analyses (WLA: *n* = 129; R11: *n* = 134; BLA: *n* = 133; L68: *n* = 128). The sample size for the statistical analysis varied for the different variables between 125 and 134 observations per layer line (130 observations on average) and was based either on individual records, or, as in the case of egg quality traits, on average values calculated over different points in time as described above. A detailed list of sample sizes separated by genotype for all variables is given in the Supplementary Material (Table S5).

The impacts of layer line, generation and their interaction on production parameters, body weight and bone traits were analysed using the GLIMMIX procedure of SAS 9.4 (SAS Institute Inc. Cary, NC, USA, 2017) according to the following model:

(1) $$\gamma_{ijkm}=\mu +LL_{i}+G_{j}+LL\times G_{ij}+S_{k}+\varepsilon_{ijkm}$$

where $\gamma_{ijkm}$ is the observation for a production parameter, body weight or bone trait, μ is the general mean, $LL_{i}$ is the fixed effect of layer line (i = 1 to 4), $G_{j}$ is the fixed effect of generation (j = 1, 2), $LL\times G_{ij}$ is the fixed effect of interaction between layer line and generation, $S_{k}$ is the random effect of sire (k = 1 to 145) and $\varepsilon_{ijkm}$ is the random error variance. Tukey’s HSD (honestly significant difference) test was performed for multiple comparisons of means. Statistical significance was set at *p* < 0.05.

To determine the association between the bone characteristics, Pearson’s correlation coefficients (r_p_) were estimated using the CORR (correlation) procedure from SAS (SAS Institute Inc. Cary, NC, USA, 2017). Results description followed recommendations by Asuero et al. [37].

An analysis of covariance was applied to study the variation of BBS considering bone morphometry, BMD and total eggshell production as covariate terms and the fixed effects of layer line, generation and significant interactions between main factors and the covariates [38]. In a backward elimination approach, the Wald F-statistics were used to determine the significance of fixed effects [39], resulting in the following model:

(2) $$\gamma_{ijklmnopq}=\mu +LL_{i}+G_{j}+LL\times G_{ij}+BMD_{k}+LL\times BMD_{ik}+G\times BMD_{jk}+LL\times G\times BMD_{ijk}+W_{l}+T_{m}+L_{n}+TEP_{o}+S_{p}+\varepsilon_{ijklmnopq}$$

where $\gamma_{ijklmnopq}$ is the observation of BBS; μ is the general mean; $LL_{i}$ is the fixed effect of layer line (i = 1 to 4); $G_{j}$ is the fixed effect of generation (j = 1, 2); $LL\times G_{ij}$ is the fixed effect of interaction between layer line and generation; $BMD_{k}$ is the effect of BMD; $LL\times BMD_{ik}$ is the effect of interaction between layer line and BMD; $G\times BMD_{jk}$ is the effect of the interaction between generation and BMD; $LL\times G\times BMD_{ijk}$ is the effect of the interaction between layer line, generation and BMD; $W_{l}$ is the effect of bone weight; $T_{m}$ is the effect of bone thickness; $L_{n}$ is the effect of bone length; $TEP_{o}$ is the effect of total egg number; $S_{p}$ is the random effect of sire (p = 1 to 145); and $\varepsilon_{ijklmnopq}$ is the random error variance.

The bone data were converted to standardised z-scores to have a standard deviation of 1.0 and a mean of 0.0. Univariate regression analyses were performed using the MIXED procedure from SAS (SAS Institute Inc. Cary, NC, USA, 2017) according to the following model:

(3) $$\gamma_{i}=\beta_{0}+\beta_{1}x_{i}+\varepsilon_{i}$$

where $\gamma_{i}$ is the BBS or the BMD; $\beta_{0}$ is the intercept; $\beta_{1}$ is the slope; $x_{i}$ is a morphometric parameter (in case of BBS analysis) or total eggshell production (in case of BMD analysis); and $\varepsilon_{i}$ is the random error variance.

Genetic parameters were estimated using ASReml 4.1 [40] according to the following animal model:

(4) $$\gamma_{ij}=\mu +A_{i}+G_{j}+\varepsilon_{ij}$$

where $\gamma_{ij}$ is the BBS or the BMD, μ is the general mean, $A_{i}$ is the random direct genetic effect of the hen, $G_{j}$ is the fixed effect of the generation (j = 1, 2) and $\varepsilon_{ij}$ is the error term. Within lines, univariate analyses were conducted to estimate the heritability of BMD and BBS. Bivariate analyses were used for estimation of genetic correlations between these traits.

## 3. Results

### 3.1. Basic Production Parameters

Table 1 summarises the least squares means and the significance of layer line, generation and their interaction for various basic production parameters. For all traits, a highly significant effect of the layer line was observed. With exception of total egg number and feed-to-egg conversion rate, the generation was also identified as a significant explanatory variable. However, only in respect to egg- and eggshell weight, the effect of layer line by generation interaction proved to be significant. Here, in line WLA heavier eggs were observed in the second generation, whereas in R11, BLA and L68, slightly higher eggshell weights were seen in the first generation. With the first eggs being laid at 20.56 (WLA) and 20.69 (BLA) weeks of age, in both high performing lines the onset of laying was significantly earlier than in their moderate performing counterparts, as L68 and R11 reached laying maturity only at 23.12 and 24.66 weeks of age, respectively. Within the 357 days lasting laying period, lines WLA and BLA achieved laying performances of 316.34 and 317.32 eggs respectively, and differed significantly from lines R11 (average of 226.25 eggs) and L68 (average of 215.94 eggs). In terms of egg weight, eggshell weight and proportion of the eggshell, the high performing genotypes showed significantly higher values than their corresponding moderate performing lines. This pattern continued for the total eggshell production. However, there was a clear ranking of genotypes, with WLA producing the largest amount of eggshell, followed by BLA, R11 and L68. The mean difference in total eggshell production within the phylogenetic groups was 821.94 g between the white-egg lines, and 812.63 g between the brown-egg lines. Despite significantly higher daily feed consumption of both high performing lines, the feed-to-egg and feed-to-eggshell conversion rates were about one third lower in both BLA and WLA hens than in their counterparts. Results on body weight development are shown in the Supplementary Material (Table S6). The fact that WLA and BLA hens were heavier at hatch reversed during rearing. The brown-egg lines were both significantly heavier during the following measurements, and at final weighing in, week 69, line L68 had the highest average body weight.

### 3.2. Bone Characteristics

The least squares means and the significance level for layer line, generation and their interaction for the examined traits of tibiotarsus and humerus are shown in Table 2. The layer line had a highly significant effect on all bone traits studied. With exception of weight and thickness in tibiotarsus, the same applies to the generation effect. The layer line by generation interaction was significant for the weight of tibiotarsus. However, post-hoc comparison did not detect any significant deviation between the two generations within lines. Hens from the brown-egg lines displayed a higher humerus BBS than the white-egg strains. For the tibiotarsus, line L68 was characterised by a high BBS, whereas the other lines differ only slightly amongst each other. Mean BMD of both bone types was significantly higher in the brown-egg lines BLA and L68, while hens of line WLA showed the lowest values. It turns out that BLA and L68 do have significantly heavier, thicker and longer bones than their white-egg-laying counterparts. Line L68 especially stands out in relation to the tibiotarsus, being the line with the highest values for all traits.

**Figure 2:**
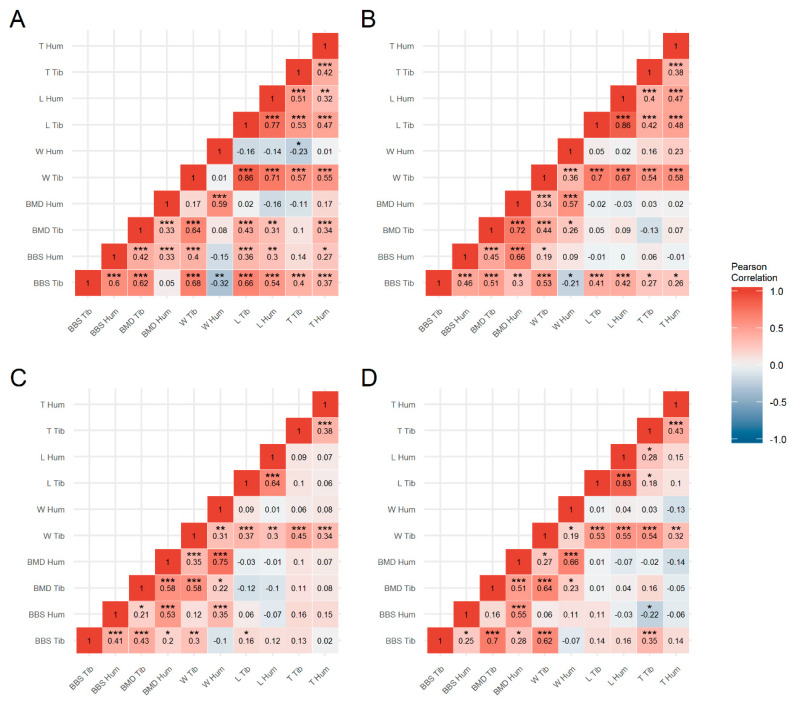
Heatmap of Pearson’s correlation coefficients between bone traits (BBS, bone breaking strength; BMD, bone mineral density; W, bone weight; L, bone length; T, bone thickness) of the tibiotarsus (Tib) and humerus (Hum) in laying hens of the genetic lines WLA (**A**), R11 (**B**), BLA (**C**) and L68 (**D**). Red indicates a positive correlation; white represents no correlation and blue represents a negative correlation. Significant correlation coefficients are marked with asterisks (* *p* < 0.05; ** *p* < 0.01; *** *p* < 0.001).

Figure 2 shows the Pearson’s correlation coefficients (r_p_) between the bone traits examined in the tibiotarsus and humerus of the genetic lines WLA (Figure 2A), R11 (Figure 2B), BLA (Figure 2C) and L68 (Figure 2D). With values varying from r_p_ = 0.43 (Figure 2C) to r_p_ = 0.70 (Figure 2D), BBS and BMD were moderately correlated for the tibiotarsus. Slightly weaker correlations ranging from r_p_ = 0.33 (Figure 2A) to r_p_ = 0.66 (Figure 2B) were observed between these traits for the humerus. Except for moderate correlations between BBS and weight in the tibiotarsus of WLA (r_p_ = 0.68, Figure 2A) and L68 (r_p_ = 0.62, Figure 2D), rather low and non-significant correlations were observed regarding BBS and morphometric traits. The same applies to the relationship between BMD and morphometry in the tibiotarsus. In both bone types, BMD and weight were moderately to strongly associated. With values from r_p_ = 0.64 (Figure 2C) up to r_p_ = 0.86 (Figure 2B), the lengths of the two different bone types were strongly associated. While the correlation of the thicknesses of the two bone types varied only between r_p_ = 0.38 (Figure 2B,C) and r_p_ = 0.43 (Figure 2D), it differed considerably for the weight. However, a general pattern of correlating characteristics that applies to all lines and/or to phylogenetic groups could not be identified.

### 3.3. Factors Affecting Bone Strength

Table 3 shows the effects of the main factors and covariates, and significant interactions on the BBS of tibiotarsus and humerus. As an extension of the basic statistical model (1), which is shown in Table 2, in model (2) different covariates are considered additionally to assessing the effects of bone morphometry and total eggshell production on BBS. The bone types studied were influenced by the layer line and the generation. However, the interaction of layer line and generation was only significant for the humerus. The analysis revealed that a high amount of the observed variance in the hens’ BBS is attributable to its BMD, as indicated by comparatively high F values being 243.50 (tibiotarsus) and 281.92 (humerus), respectively. The bone types differed with regard to the influence of morphometry on fracture strength, as an effect of bone thickness and length was only observed for the tibiotarsus, while bone weight did not play a role at all. Within lines, an effect of total eggshell production on the BBS was also not detectable, leading to the assumption that this variable does not contribute to the variance in BBS within the lines studied.

**Figure 3:**
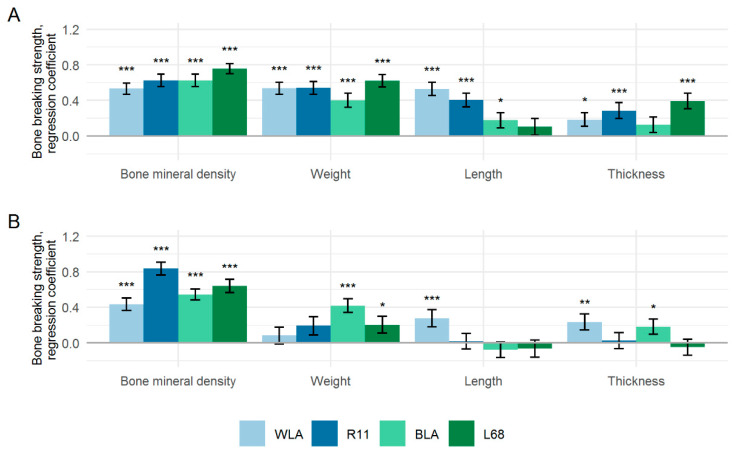
Standardised regression coefficients (β) ± standard errors of regression of bone mineral density, bone weight, bone length and bone thickness pertaining univariately to the bone breaking strengths of the tibiotarsus (**A**) and humerus (**B**) in four different chicken layer lines (WLA, R11, BLA, L68). Significant regression coefficients are marked with asterisks (* *p* < 0.05; ** *p* < 0.01; *** *p* < 0.001).

Figure 3 shows the regression coefficients (β) between standardised bone traits; i.e., between BBS and BMD or BBS and morphometric traits. Highly significant regression coefficients varying from β = 0.53 (WLA) to β = 0.76 (L68) among the layer lines illustrate that the BMD is strongly associated with the variability of the tibiotarsal BBS (Figure 3A). On average across lines, a change in BMD by one standard deviation results in a 0.64 standard deviation increase in BBS. For the humerus, regression coefficients between BBS and BMD were proved to be significant and BMD was detected as primary explanatory variable of the BBS, although coefficients widely ranged from β = 0.43 (WLA) to C = 0.84 (R11) among the lines (Figure 3B). Line R11 stands out in this regard, while the average coefficient among the layer lines was β = 0.61. If bone weight is considered as an explanatory variable, the analysis attributed a relatively large and highly significant effect on BBS, at least for the tibiotarsus, where the average coefficient was β = 0.53. In contrast, the coefficients vary greatly in the case of the humerus, resulting in an average value of β = 0.22. A significant effect of bone weight on humeral BBS was only seen in the brown-egg lines BLA and L68. With average values of β = 0.31 and β = 0.25 respectively, the length and thickness of the tibiotarsus were only weakly correlated with BBS. However, these correlations were significant for the majority of lines. For the length (β = 0.04) and thickness (β = 0.09) of the humerus, a rather weak influence was observed, it being only occasionally significant.

**Figure 4:**
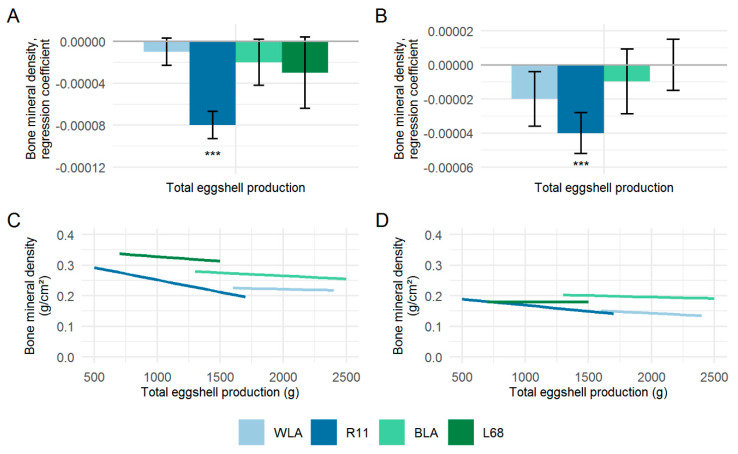
Regression coefficients (β) ± standard errors of regression of total eggshell production pertaining to the bone mineral densities of the tibiotarsus (**A**) and humerus (**B**), and the effect of total eggshell production on the bone mineral densities of the tibiotarsus (**C**) and humerus (**D**) in four different chicken layer lines (WLA, R11, BLA, L68). Significant regression coefficients are marked with asterisks (*** *p* < 0.001).

Figure 4 shows the regression coefficients (β) of the total eggshell production in relation to the BMD of the tibiotarsus (Figure 4A) and humerus (Figure 4B). Overall, rather low negative regression coefficients were obtained for the two bone types, averaging β = −3.5 × 10^−5^ (tibiotarsus) and β = −1.7 × 10^−5^ (humerus) respectively. In both bone types, regression coefficients were only significant in the low performing white-egg line R11. However, other significant relationships between BMD and eggshell production, could not be observed. Figure 4C (tibiotarsus) and Figure 4D (humerus) show the trend of BMD with increasing total eggshell production within the chicken lines studied. Considering the range of variation in BMD (see Table 2), an increase of total eggshell production appears to have only limited effects on BMD, especially for hens of layer lines WLA, BLA and L68.

### 3.4. Genetic Parameters

Results of heritability (h²) estimates for BBS and BMD and the genetic correlations (r_g_) between these traits are shown in Table 4. Due to the lack of convergence of the model, no h² estimations were possible for the BMD of the tibiotarsus of line R11 and for the humerus of the WLA line. Accordingly, the genetic correlation coefficients could not be estimated in these cases. The h² values estimated for BBS vary rather strongly among lines. In case of the tibiotarsus, for example, they vary from h² = 0.17 (BLA) to h² = 0.58 (WLA). A similar situation was found for the humerus, for which the values range from h² = 0.26 (WLA) to h² = 0.50 (BLA). The h² estimation for the BMD values of the tibiotarsus and humerus resulted in similarly fluctuating values, among which the line WLA stands out at h² = 0.75 for the tibiotarsus and h² = 0.73 for the line R11. Estimated r_g_ coefficients suggest a moderate to close genetic relationship between BBS and BMD, except for the tibiotarsus of the BLA line, where it was estimated to be only r_g_ = 0.16.

## 4. Discussion

The aim of the current study was to analyse the relationship among bone stability traits and egg production in four phylogenetically divergent layer lines differing in their performance levels. The phylogenetic divergence provides insights into the impacts of different breeding histories, which may have affected bone stability before commercial poultry breeding began; the difference in performance level may provide a hint as to the effect of selection for high egg yield within groups of brown and white-egg layer lines.

### 4.1. Phenotypic Characterization

We observed significant differences among the layer lines regarding all examined production parameters, which are consistent with previous reports [21,33]. Both high performing lines were superior to their counterparts, which was expected, as commercial lines have long been selected for age at laying maturity, peak production and laying persistency [41,42]. The results on body weight and feed efficiency clearly reflect the efforts made toward improving feed conversion [1,42]. Our results indicated significantly higher amounts of calcium required for eggshell formation in the high performing genotypes that can compensate for this by stimulated bone resorption and/or better intestinal calcium absorption. The latter should probably be reflected in an increased expression of epithelial calcium transporting proteins [43]. However, further assumptions on this require a detailed investigation of calcium homeostasis, which could be addressed in further studies.

In accordance with Riczu et al. [44] and Habig et al. [21] our results on bone measurements revealed a strong phylogenetic divergence between brown and white-egg layer lines. Consistent performance-related differences were only found for the BMD, as within the phylogenetic groups the moderate performers possessed a significantly higher BMD. With regard to the BBS, we observed rather inconsistent results. Nevertheless, our results confirmed the tendency for the two high performing genotypes to have lower bone stability [21]. Contrary to the findings of Bishop et al. [19], we did not observe a strong correlation between the tibiotarsal and the humeral BBS, which was evident across all lines. Rather, the correlation varied depending on the layer line. Taken together, the results on the production parameters and bone characteristics reflect remarkably phenotypic differences among the layer lines.

### 4.2. Determinants of Bone Stability

The analyses clearly turned out that the BMD significantly contributes to the variation in BBS, which is consistent with an earlier study on White Leghorn hens [45]. According to our observations, this can probably be extended to laying hens in general. Our findings are in line with those from others who have associated BMD with biomechanical strength, which is why it plays an important role in osteoporosis [4,46]. A histological differentiation of cortical and medullary bone tissue, e.g., by means of quantitative computed tomography [47], would be helpful for a more detailed insight into the components affecting bone stability.

Since the bone stability and the whole bone properties are inseparably linked by the bone’s architecture and geometry [48,49], morphometric bone traits were considered as covariates of the BBS. Only in the tibiotarsus, could a rather small effect of morphometry on fracture strength be accounted. However, this is marginal, given the tremendous influence of the BMD. Contrary to our assumption, bone weight did not contribute to the variance in BBS at all. This could be because BMD is already considered in the statistical model and is strongly correlated with bone weight and/or because BMD indirectly integrates bone dimensions as it relates to the scanned bone area [50].

Interestingly, the total eggshell production had no significant effect on BBS or BMD of tibiotarsus and humerus. This was not expected, because although this assumption is controversially discussed, the level of egg production is frequently claimed to be detrimental [51]. Considering our findings and those from other studies that have reported an absence of relationship between bone stability and egg production [47,51,52,53,54], evidence for a strong association within chicken lines seems rather questionable. However, if we only compare lines differing in performance level, our results would suggest that osteoporosis is mainly caused by a high laying rate, supporting earlier conclusions that differences of bone quality characteristics between genotypes should not be oversimplified [54].

We observed individuals that produced high amounts of eggshells and at the same time had high BBS values, which may indicate that high laying rate and good bone quality are not mutually exclusive. However, some studies pointed to the laying persistency causing continuous degradation of structural bone tissue, rather than the precise number of eggs [4,7,54]. This likely applies to our moderately performing lines, since their reduced egg number necessarily involved periods of laying inactivity, during which they were able to regenerate. An adverse effect of a premature onset of laying, at which the ossification is possibly not yet sufficiently complete, was also suggested [3,53]. Possibly, these two factors will ultimately have a combined effect.

### 4.3. Genetic Perspectives

The results regarding genetic parameters indicate a rather close genetic relationship between BBS and BMD in all layer lines, completing our findings from the phenotypic analyses. With an average h² estimate of 0.39, we can confirm the moderate inheritance of BBS [19]. However, the h² estimates were quite variable and considerable differences among lines were observed. This might reflect diverse genetic composition or distinct breeding history of the lines studied [30,31]. Given the large individual variation in bone characteristics and the implied inherited component of susceptibility to osteoporosis, the problem of skeletal damage is assumed to be alleviated by genetic selection [18,19,22,54]. Our results support this assumption based on the h² estimates of the BBS presented. The current study emphasizes the great importance of animal breeding, offering promising possibilities to counteract the loss of bone strength. At that, the eggshell quality must continue to be considered in the selection index to improve bone stability without compromising eggshell quality.

## 5. Conclusions

In this study, we analysed the variation of bone breaking strength (BBS) within phylogenetically divergent chicken layer lines, differing in their levels of egg production. The current results support earlier findings that bone mineral density (BMD) is of particular importance for the BBS. Results do not provide evidence of a strong association between the total eggshell production and bone stability traits within the genetic lines studied. Finally, the estimation of genetic parameters revealed an inherited component of BBS and BMD. A rather weak correlation between laying performance and bone stability was observed, opening up the possibility to select for improved bone stability without adverse effects on laying performance. Due to the line specificity in the various phenotypic characteristics, generalised statements about a possible superiority of a certain phylogenetic group or performance level are not justified.

## Figures and Tables

**Table 1:** Least squares means ± standard errors for production parameters for the effect of layer line (LL), generation (Gen) and their interaction, and the significance levels of the effects.

Effect	Laying Maturity (Weeks)	Total Number of Eggs ^1^	Egg Weight (g) ^2^	Eggshell Weight (g) ^2^	Eggshell Proportion (%) ^2^	Total Eggshell Production (g) ^3^	Daily Feed Consumption (g)	Feed-to-Egg Conversion Rate	Feed-to-Eggshell Conversion Rate
**Layer line (LL)**	<0.0001	<0.0001	<0.0001	<0.0001	<0.0001	<0.0001	<0.0001	<0.0001	<0.0001
WLA	20.56 ± 0.15 ^c^	316.34 ± 2.57 ^a^	57.63 ± 0.41^b^	6.38 ± 0.04 ^a^	11.10 ± 0.06 ^a^	2019.89 ± 16.23 ^a^	93.82 ± 0.97 ^b^	1.84 ± 0.02 ^c^	16.64 ± 0.29 ^d^
R11	24.66 ± 0.15 ^a^	226.25 ± 2.53 ^b^	51.54 ± 0.41^c^	5.29 ± 0.04 ^c^	10.28 ± 0.06 ^b^	1197.95 ± 16.00 ^c^	79.58 ± 0.96 ^c^	2.47 ± 0.02 ^b^	24.14 ± 0.29 ^b^
BLA	20.69 ± 0.15 ^c^	317.32 ± 2.55 ^a^	60.09 ± 0.41^a^	6.14 ± 0.04 ^b^	10.25 ± 0.06 ^b^	1950.23 ± 16.08 ^b^	102.46 ± 0.98 ^a^	1.92 ± 0.02 ^c^	18.80 ± 0.29 ^c^
L68	23.12 ± 0.15 ^b^	215.94 ± 2.57 ^c^	53.05 ± 0.41^c^	5.29 ± 0.04 ^c^	9.95 ± 0.06 ^c^	1137.60 ± 16.27 ^d^	91.32 ± 0.98 ^b^	2.88 ± 0.02 ^a^	29.04 ± 0.29 ^a^
**Generation (Gen)**	<0.0001	0.8384	0.0032	<0.0001	<0.0001	<0.0001	0.0017	0.3673	<0.0001
Gen1	21.81 ± 0.11	269.23 ± 1.82	54.97 ± 0.29	5.94 ± 0.03	10.80 ± 0.05	1619.31 ± 11.53	90.25 ± 0.69	2.27 ± 0.02	21.14 ± 0.21
Gen2	22.70 ± 0.11	268.70 ± 1.79	56.19 ± 0.29	5.62 ± 0.03	9.99 ± 0.05	1533.53 ± 11.30	93.33 ± 0.68	2.29 ± 0.02	23.17 ± 0.20
**LL × Gen**	0.4859	0.2202	0.0470	0.0028	0.4750	0.1823	0.6130	0.9523	0.2921
WLA × Gen1	20.01 ± 0.22	319.87 ± 3.64	56.16 ± 0.59 ^b^	6.41 ± 0.06 ^a^	11.42 ± 0.09	2048.99 ± 22.99	91.57 ± 1.38	1.82 ± 0.03	16.00 ± 0.41
WLA × Gen2	21.10 ± 0.22	312.80 ± 3.63	59.11 ± 0.58 ^a^	6.37 ± 0.06 ^a^	10.78 ± 0.09	1990.80 ± 22.91	96.06 ± 1.37	1.86 ± 0.03	17.27 ± 0.41
R11 × Gen1	24.42 ± 0.21	224.59 ± 3.58	51.44 ± 0.58 ^c^	5.51 ± 0.06 ^c^	10.71 ± 0.09	1237.57 ± 22.66	78.85 ± 1.36	2.47 ± 0.03	23.09 ± 0.41
R11 × Gen2	24.90 ± 0.22	227.92 ± 3.64	51.64 ± 0.58 ^c^	5.08 ± 0.06 ^d^	9.84 ± 0.09	1158.33 ± 22.58	80.30 ± 1.37	2.47 ± 0.03	25.20 ± 0.41
BLA × Gen1	20.20 ± 0.22	319.53 ± 3.65	59.28 ± 0.59 ^a^	6.33 ± 0.06 ^a^	10.70 ± 0.09	2023.14 ± 23.10	101.39 ± 1.41	1.91 ± 0.04	17.85 ± 0.42
BLA × Gen2	21.19 ± 0.21	315.12 ± 3.56	60.91 ± 0.58 ^a^	5.95 ± 0.06 ^b^	9.80 ± 0.09	1877.31 ± 22.38	103.53 ± 1.35	1.93 ± 0.03	19.74 ± 0.40
L68 × Gen1	22.62 ± 0.22	212.90 ± 3.70	53.00 ± 0.59 ^c^	5.49 ± 0.06 ^c^	10.37 ± 0.09	1167.53 ± 23.45	89.21 ± 1.41	2.86 ± 0.04	27.62 ± 0.42
L68 × Gen2	23.62 ± 0.22	218.99 ± 3.58	53.10 ± 0.58 ^c^	5.06 ± 0.06 ^d^	9.54 ± 0.09	1107.67 ± 22.56	93.43 ± 1.36	2.89 ± 0.03	30.46 ± 0.40

^a,b,c,d^ Means with different letters within an effect differ significantly (Tukey’s HSD-test, *p* < 0.05). ^1^ Laid between weeks 18 and 68 (357 days). ^2^ Mean value of the eggs laid from 18 to 68 weeks of age. ^3^ Product of total number of eggs and mean eggshell weight.

**Table 2:** Least squares means ± standard error for characteristics of tibiotarsus and humerus for the effect of layer line (LL), generation (Gen) and their interaction and the significance levels for the effects.

Effect	Tibiotarsus					Humerus				
	Bone Breaking Strength (N)	Bone Mineral Density (g/cm²)	Weight (g)	Length (mm)	Thickness (mm)	Bone Breaking Strength (N)	Bone Mineral Density (g/cm²)	Weight (g)	Length (mm)	Thickness (mm)
**Layer line (LL)**	<0.0001	<0.0001	<0.0001	<0.0001	<0.0001	<0.0001	<0.0001	<0.0001	<0.0001	<0.0001
WLA	137.34 ± 3.62 ^b,c^	0.211 ± 0.005 ^d^	8.12 ± 0.11 ^c^	116.78 ± 0.44 ^c^	5.27 ± 0.04 ^c^	90.81 ± 3.43 ^c^	0.136 ± 0.003 ^d^	3.76 ± 0.10 ^c^	75.81 ± 0.25 ^c^	5.33 ± 0.03 ^c^
R11	149.40 ± 3.54 ^b^	0.231 ± 0.005 ^c^	8.23 ± 0.11 ^c^	116.63 ± 0.44 ^c^	5.17 ± 0.04 ^c^	109.94 ± 3.40 ^b^	0.156 ± 0.003 ^c^	4.07 ± 0.10 ^c^	75.38 ± 0.25 ^c^	5.19 ± 0.03 ^d^
BLA	124.23 ± 3.58 ^c^	0.265 ± 0.005 ^b^	10.90 ± 0.11 ^b^	119.41 ± 0.44 ^b^	5.94 ± 0.04 ^b^	138.64 ± 3.40 ^a^	0.197 ± 0.003 ^a^	6.53 ± 0.10 ^a^	79.69 ± 0.25 ^a^	5.59 ± 0.03 ^b^
L68	211.57 ± 3.61 ^a^	0.327 ± 0.005 ^a^	12.03 ± 0.11 ^a^	121.37 ± 0.44 ^a^	6.19 ± 0.04 ^a^	146.02 ± 3.45 ^a^	0.180 ± 0.003 ^b^	4.93 ± 0.10 ^b^	76.85 ± 0.25 ^b^	5.71 ± 0.03 ^a^
**Generation (Gen)**	<0.0001	0.0369	0.7077	0.0002	0.1231	<0.0001	<0.0001	<0.0001	0.0021	0.0327
Gen 1	139.23 ± 2.55	0.264 ± 0.004	9.80 ± 0.08	117.74 ± 0.31	5.61 ± 0.03	111.27 ± 2.43	0.174 ± 0.002	5.60 ± 0.07	76.54 ± 0.18	5.42 ± 0.02
Gen 2	172.04 ± 2.53	0.253 ± 0.004	9.84 ± 0.07	119.36 ± 0.31	5.67 ± 0.03	131.43 ± 2.40	0.161 ± 0.002	4.05 ± 0.07	77.32 ± 0.18	5.49 ± 0.02
**LL × Gen**	0.0925	0.1725	0.0155	0.0892	0.4924	0.5249	0.8472	0.5376	0.2413	0.0665
WLA × Gen1	113.51 ± 5.10	0.210 ± 0.007	7.84 ± 0.15 ^c^	115.03 ± 0.62	5.20 ± 0.05	77.13 ± 4.85	0.142 ± 0.004	4.51 ± 0.15	75.00 ± 0.36	5.27 ± 0.04
WLA × Gen2	161.16 ± 5.15	0.214 ± 0.007	8.40 ± 0.15 ^c^	118.54 ± 0.62	5.35 ± 0.05	104.48 ± 4.85	0.131 ± 0.004	3.02 ± 0.15	76.61 ± 0.36	5.40 ± 0.04
R11 × Gen1	132.92 ± 5.02	0.233 ± 0.007	8.25 ± 0.15 ^c^	116.22 ± 0.61	5.15 ± 0.05	103.33 ± 4.78	0.162 ± 0.004	4.89 ± 0.15	74.99 ± 0.35	5.21 ± 0.04
R11 × Gen2	165.87 ± 5.01	0.229 ± 0.007	8.21 ± 0.15 ^c^	117.05 ± 0.62	5.20 ± 0.05	116.55 ± 4.83	0.151 ± 0.004	3.24 ± 0.14	75.77 ± 0.35	5.18 ± 0.04
BLA × Gen1	112.38 ± 5.12	0.275 ± 0.007	11.10 ± 0.15 ^b^	119.07 ± 0.63	5.93 ± 0.05	127.87 ± 4.89	0.205 ± 0.004	7.20 ± 0.15	79.44 ± 0.36	5.59 ± 0.04
BLA × Gen2	136.07 ± 5.01	0.255 ± 0.007	10.71 ± 0.15 ^b^	119.75 ± 0.61	5.94 ± 0.05	149.40 ± 4.72	0.190 ± 0.004	5.86 ± 0.14	79.94 ± 0.35	5.59 ± 0.04
L68 × Gen1	198.11 ± 5.20	0.338 ± 0.007	12.02 ± 0.15 ^a^	120.62 ± 0.63	6.18 ± 0.05	136.75 ± 4.94	0.188 ± 0.004	5.79 ± 0.15	76.74 ± 0.36	5.62 ± 0.04
L68 × Gen2	225.03 ± 5.03	0.315 ± 0.007	12.04 ± 0.15 ^a^	122.12 ± 0.62	6.20 ± 0.05	155.29 ± 4.83	0.171 ± 0.004	4.07 ± 0.14	76.97 ± 0.35	5.79 ± 0.04

^a,b,c,d^ Means with different letters within an effect differ significantly (Tukey’s HSD-test, *p* < 0.05).

**Table 3:** The effects of layer line (LL), generation (Gen), bone mineral density (BMD), bone weight, bone thickness, bone length, total eggshell production and significant interactions on bone breaking strengths of the tibiotarsus and humerus in laying hens.

Effect	Tibiotarsus		Humerus	
	*F* Value	*p*-Value	*F* Value	*p*-Value
Layer line (LL)	9.10	<0.0001	8.13	<0.0001
Generation (Gen)	13.22	0.0003	8.92	0.0030
LL × Gen	1.75	0.1568	5.58	0.0009
Bone mineral density (BMD)	243.50	<0.0001	281.92	<0.0001
BMD × LL	24.71	<0.0001	10.53	<0.0001
BMD × Gen	33.96	<0.0001	26.59	<0.0001
BMD × LL × Gen	2.08	0.1025	7.23	<0.0001
Weight	0.00	0.9927	3.42	0.0654
Thickness	23.33	<0.0001	0.62	0.4319
Length	10.90	0.0011	0.09	0.7660
Total eggshell production ^1^	0.13	0.7196	0.07	0.7879

^1^ Total eggshell production = number of eggs × eggshell weight.

**Table 4:** Heritability (h²; ± standard error) and genetic correlation (r_g_; ± standard error) estimated for bone breaking strength (BBS) and bone mineral density (BMD) of tibiotarsus and humerus in four chicken layer lines.

Layer Line	Tibiotarsus			Humerus		
	h² BBS	h² BMD	r_g_	h² BBS	h² BMD	r_g_
WLA	0.58 ± 0.23	0.75 ± 0.23	0.93 ± 0.23	0.26 ± 0.22	N.A.	N.A.
R11	0.29 ± 0.22	N.A.	N.A.	0.40 ± 0.20	0.73 ± 0.21	0.81 ± 0.18
BLA	0.17 ± 0.19	0.55 ± 0.20	0.16 ± 0.37	0.50 ± 0.26	0.25 ± 0.17	0.54 ± 0.30
L68	0.46 ± 0.23	0.51 ± 0.23	0.74 ± 0.17	0.44 ± 0.21	0.46 ± 0.25	0.79 ± 0.18

N.A., not analysable.

## Supplementary Materials

The following are available online at https://www.mdpi.com/2076-2615/10/5/850/s1. Table S1: Light program and mean climatic conditions, Table S2: Calculated nutrient compositions of the diets fed to the chicks during the first and second generations, respectively, and of the diet fed to the pullets during both generations, Table S3: Ingredients and analysed nutrient compositions of the experimental layer diets of the first and second generations, Table S4: The effects of layer line, diet and their interaction on the observed bone traits and basic production parameters calculated within generations, Table S5: Sample sizes for the analysis, Table S6: Least squares means ± standard errors and level of significance for body weight measured at hatching and different weeks of age under the effect of layer line (LL), generation (Gen) and their interaction.
