# Correction: Jansen, S., et al. Relationship between Bone Stability and Egg Production in Genetically Divergent Chicken Layer Lines. *Animals* 2020, *10*, 850

**DOI:** 10.3390/ani10122355

**Published:** 2020-12-09

**Authors:** Simon Jansen, Ulrich Baulain, Christin Habig, Annett Weigend, Ingrid Halle, Armin Manfred Scholz, Henner Simianer, Ahmad Reza Sharifi, Steffen Weigend

**Affiliations:** 1 Institute of Farm Animal Genetics, Friedrich-Loeffler-Institut, 31535 Neustadt, Germany; ulrich.baulain@fli.de (U.B.); christin.habig@fli.de (C.H.); annett.weigend@fli.de (A.W.); steffen.weigend@fli.de (S.W.); 2 Institute of Animal Nutrition, Friedrich-Loeffler-Institut, 38116 Braunschweig, Germany; ingrid.halle@fli.de; 3 Livestock Center of the Faculty of Veterinary Medicine, Ludwig-Maximilians-University Munich, 85764 Oberschleissheim, Germany; armin.scholz@lvg.vetmed.uni-muenchen.de; 4 Animal Breeding and Genetics Group, Department of Animal Sciences, University of Göttingen, 37075 Göttingen, Germany; hsimian@gwdg.de (H.S.); rsharif@gwdg.de (A.R.S.); 5 Center for Integrated Breeding Research, University of Göttingen, 37075 Göttingen, Germany

The authors wish to make the following corrections to this paper [1]:

The body weight given as the weight of the 35th week of age is in fact the weight of the 49th week of age. However, the data presented are correct and the changes do not alter their interpretation.

## Main Body Paragraphs Correction

There was an error in the original article. On page 4, Section *2.3. Experimental Procedure*, 1st paragraph, the sentence:

“Body weight (g) was measured at hatch and during the experimental period (at week 21, 25, 35 and 69) using a digital table scale (CPA 16001S, Sartorius, Göttingen, Germany) with a weighing accuracy of 0.1 g.”

should be

“Body weight (g) was measured at hatch and during the experimental period (at week 21, 25, 49 and 69) using a digital table scale (CPA 16001S, Sartorius, Göttingen, Germany) with a weighing accuracy of 0.1 g.”

## Figures/Tables Correction

Due to the mistake mentioned above, we need to make the following changes to figures and tables:

Replace Figure 1:

**Figure 1:**
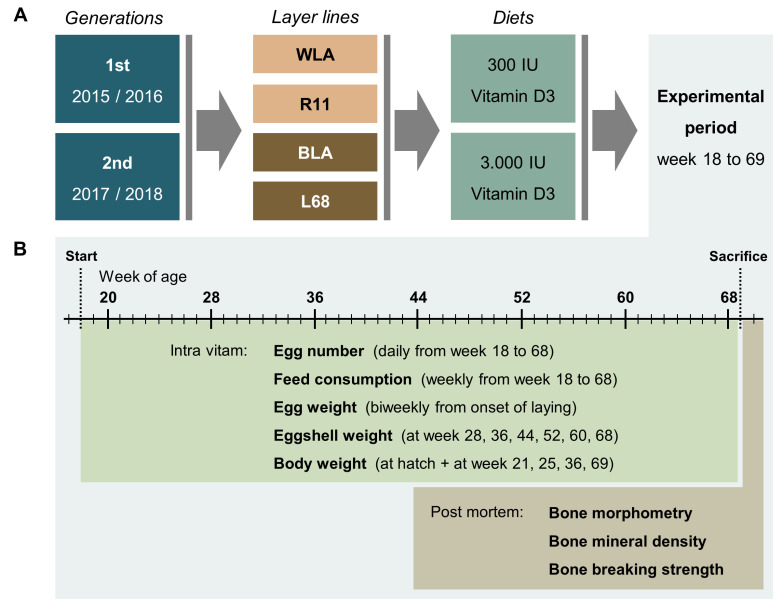
Schematic illustration of the experimental setup (**A**) and related data collection (**B**). In two consecutive generations, four chicken layer lines were allocated to a diet containing either 300 or 3000 IU of vitamin D3. During the experimental period, data on egg number, egg quality, feed consumption, and body weight were collected as indicated. Post mortem, bone morphometry, bone mineral density, and bone breaking strength were assessed.

With new Figure 1 below:

**Figure 1:**
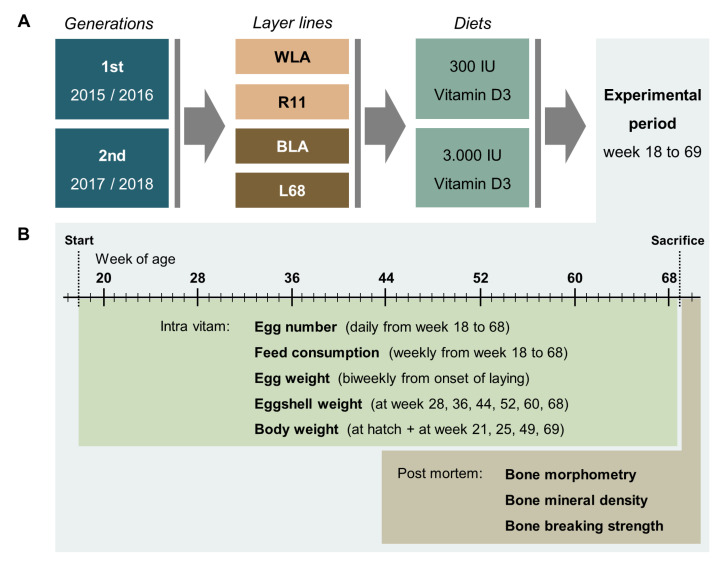
Schematic illustration of the experimental setup (**A**) and related data collection (**B**). In two consecutive generations, four chicken layer lines were allocated to a diet containing either 300 or 3000 IU of vitamin D3. During the experimental period, data on egg number, egg quality, feed consumption, and body weight were collected as indicated. Post mortem, bone morphometry, bone mineral density, and bone breaking strength were assessed.

## Change in Supplementary File

Replace Table S5:

**Table S5:** Sample sizes for the analysis.

Variable	Total	Layer Line			
		WLA	R11	BLA	L68
Laying maturity	524	129	134	133	128
Total number of eggs	524	129	134	133	128
Egg weight	524	129	134	133	128
Eggshell weight	524	129	134	133	128
Eggshell proportion	524	129	134	133	128
Total eggshell production	524	129	134	133	128
Daily feed consumption	513	128	131	129	125
Feed-to-egg-conversion rate	513	128	131	129	125
Feed-to-eggshell conversion rate	513	128	131	129	125
Bone breaking strength Tibiotarsus	518	126	134	131	127
Bone mineral density Tibiotarsus	524	129	134	133	128
Weight Tibiotarsus	524	129	134	133	128
Length Tibiotarsus	524	129	134	133	128
Thickness Tibiotarsus	524	129	134	133	128
Bone breaking strength Humerus	516	128	131	132	125
Bone mineral density Humerus	519	129	134	128	128
Weight Humerus	521	127	134	132	128
Length Humerus	523	129	134	132	128
Thickness Humerus	523	129	134	132	128
Body weight at hatch	523	129	133	133	128
Body weight at week 21	524	129	134	133	128
Body weight at week 25	524	129	134	133	128
Body weight at week 35	524	129	134	133	128
Body weight at week 69	524	129	134	133	128

With new Table S5:

**Table S5:** Sample sizes for the analysis.

Variable	Total	Layer Line			
		WLA	R11	BLA	L68
Laying maturity	524	129	134	133	128
Total number of eggs	524	129	134	133	128
Egg weight	524	129	134	133	128
Eggshell weight	524	129	134	133	128
Eggshell proportion	524	129	134	133	128
Total eggshell production	524	129	134	133	128
Daily feed consumption	513	128	131	129	125
Feed-to-egg-conversion rate	513	128	131	129	125
Feed-to-eggshell conversion rate	513	128	131	129	125
Bone breaking strength Tibiotarsus	518	126	134	131	127
Bone mineral density Tibiotarsus	524	129	134	133	128
Weight Tibiotarsus	524	129	134	133	128
Length Tibiotarsus	524	129	134	133	128
Thickness Tibiotarsus	524	129	134	133	128
Bone breaking strength Humerus	516	128	131	132	125
Bone mineral density Humerus	519	129	134	128	128
Weight Humerus	521	127	134	132	128
Length Humerus	523	129	134	132	128
Thickness Humerus	523	129	134	132	128
Body weight at hatch	523	129	133	133	128
Body weight at week 21	524	129	134	133	128
Body weight at week 25	524	129	134	133	128
Body weight at week 49	524	129	134	133	128
Body weight at week 69	524	129	134	133	128

And replace Table S6:

**Table S6:** Least squares means ± standard errors and level of significance for body weight measured at hatching, and different weeks of age under the effect of layer line (LL), generation (Gen), and their interaction.

Effect	Body Weight (g)						
	Hatch	Week 21		Week 25	Week 35		Week 69
Layer line (LL)							
WLA	38.35 ± 0.37 ^a^	1420.02 ± 15.79 ^b^		1468.38 ± 16.19 ^b^	1497.54 ± 20.46 ^b^		1504.23 ± 22.26 ^c^
R11	33.17 ± 0.36 ^c^	1040.84 ± 15.60 ^c^		1236.40 ± 15.99 ^c^	1309.28 ± 20.21 ^c^		1362.79 ± 21.99 ^d^
BLA	39.35 ± 0.37 ^a^	1584.15 ± 15.71 ^a^		1663.55 ± 16.11 ^a^	1821.81 ± 20.34 ^a^		1838.10 ± 22.13 ^b^
L68	34.84 ± 0.37 ^b^	1568.91 ± 15.81 ^a^		1714.92 ± 16.21 ^a^	1837.91 ± 20.48 ^a^		1923.44 ± 22.29 ^a^
Generation (Gen)							
Gen 1	35.86 ± 0.26	1379.01 ± 11.17		1485.67 ± 11.45	1567.49 ± 14.47		1616.21 ± 15.76
Gen 2	37.00 ± 0.26	1427.95 ± 11.07		1555.96 ± 11.36	1665.79 ± 14.34		1698.07 ± 15.59
LL × Gen							
WLA × Gen1	37.77 ± 0.52	1376.72 ± 22.36		1415.84 ± 22.91	1460.33 ± 28.96		1443.48 ± 31.51
WLA × Gen2	38.93 ± 0.52	1463.31 ± 22.32		1520.93 ± 22.88	1534.75 ± 28.91		1564.98 ± 31.45
R11 × Gen1	32.64 ± 0.51	1027.33 ± 21.99		1222.77 ± 22.53	1284.20 ± 28.49		1338.12 ± 31.02
R11 × Gen2	33.69 ± 0.52	1054.36 ± 22.14		1250.04 ± 22.71	1334.37 ± 28.66		1387.45 ± 31.17
BLA × Gen1	38.84 ± 0.52	1549.66 ± 22.41		1627.91 ± 22.97	1767.90 ± 29.03		1804.33 ± 31.60
BLA × Gen2	39.87 ± 0.51	1618.63 ± 22.02		1699.19 ± 22.59	1875.73 ± 28.50		1871.86 ± 30.98
L68 × Gen1	34.18 ± 0.52	1562.32 ± 22.61		1676.16 ± 23.16	1757.53 ± 29.31		1878.89 ± 31.92
L68 × Gen2	35.51 ± 0.51	1575.50 ± 22.11		1753.67 ± 22.68	1918.30 ± 28.62		1968.00 ± 31.12
ANOVA significance level (*p* value)							
	Layer line		Generation			LL × Gen	
Hatch	<0.0001		0.0019			0.9908	
Week 21	<0.0001		0.0020			0.3097	
Week 25	<0.0001		<0.0001			0.3907	
Week 35	<0.0001		<0.0001			0.2486	
Week 69	0.0003		<0.0001			0.6892	

Means within a column with different letters differ significantly (Tukey’s HSD-Test, *p* < 0.05).

With new Table S6:

**Table S6:** Least squares means ± standard errors and level of significance for body weight measured at hatching, and different weeks of age under the effect of layer line (LL), generation (Gen), and their interaction.

Effect	Body Weight (g)						
	Hatch	Week 21		Week 25	Week 49		Week 69
Layer line (LL)							
WLA	38.35 ± 0.37 ^a^	1420.02 ± 15.79 ^b^		1468.38 ± 16.19 ^b^	1497.54 ± 20.46 ^b^		1504.23 ± 22.26 ^c^
R11	33.17 ± 0.36 ^c^	1040.84 ± 15.60 ^c^		1236.40 ± 15.99 ^c^	1309.28 ± 20.21 ^c^		1362.79 ± 21.99 ^d^
BLA	39.35 ± 0.37 ^a^	1584.15 ± 15.71 ^a^		1663.55 ± 16.11 ^a^	1821.81 ± 20.34 ^a^		1838.10 ± 22.13 ^b^
L68	34.84 ± 0.37 ^b^	1568.91 ± 15.81 ^a^		1714.92 ± 16.21 ^a^	1837.91 ± 20.48 ^a^		1923.44 ± 22.29 ^a^
Generation (Gen)							
Gen 1	35.86 ± 0.26	1379.01 ± 11.17		1485.67 ± 11.45	1567.49 ± 14.47		1616.21 ± 15.76
Gen 2	37.00 ± 0.26	1427.95 ± 11.07		1555.96 ± 11.36	1665.79 ± 14.34		1698.07 ± 15.59
LL × Gen							
WLA × Gen1	37.77 ± 0.52	1376.72 ± 22.36		1415.84 ± 22.91	1460.33 ± 28.96		1443.48 ± 31.51
WLA × Gen2	38.93 ± 0.52	1463.31 ± 22.32		1520.93 ± 22.88	1534.75 ± 28.91		1564.98 ± 31.45
R11 × Gen1	32.64 ± 0.51	1027.33 ± 21.99		1222.77 ± 22.53	1284.20 ± 28.49		1338.12 ± 31.02
R11 × Gen2	33.69 ± 0.52	1054.36 ± 22.14		1250.04 ± 22.71	1334.37 ± 28.66		1387.45 ± 31.17
BLA × Gen1	38.84 ± 0.52	1549.66 ± 22.41		1627.91 ± 22.97	1767.90 ± 29.03		1804.33 ± 31.60
BLA × Gen2	39.87 ± 0.51	1618.63 ± 22.02		1699.19 ± 22.59	1875.73 ± 28.50		1871.86 ± 30.98
L68 × Gen1	34.18 ± 0.52	1562.32 ± 22.61		1676.16 ± 23.16	1757.53 ± 29.31		1878.89 ± 31.92
L68 × Gen2	35.51 ± 0.51	1575.50 ± 22.11		1753.67 ± 22.68	1918.30 ± 28.62		1968.00 ± 31.12
ANOVA significance level (*p* value)							
	Layer line		Generation			LL × Gen	
Hatch	<0.0001		0.0019			0.9908	
Week 21	<0.0001		0.0020			0.3097	
Week 25	<0.0001		<0.0001			0.3907	
Week 49	<0.0001		<0.0001			0.2486	
Week 69	0.0003		<0.0001			0.6892	

Means within a column with different letters differ significantly (Tukey’s HSD-Test, *p* < 0.05).

The authors would like to apologize for any inconvenience caused to the readers by these changes.
