# Supplementary material for: Relationship between Bone Stability and Egg Production in Genetically Divergent Chicken Layer Lines
Source: Animals (Basel). 2020 May 14;10(5):850. doi: 10.3390/ani10050850 (PMC7278460; doi:10.3390/ani10050850)
Supplement: Supplementary file 1 [file animals-10-00850-s001.zip › Supplement_TableS1.pdf]

Supplementary Material

Table S1. Light program and mean climatic conditions.

Week of age	Time		Day length (h)	Light intensity (Lux)	Temperature (°C)	Relative humidity (%)
	Start	End				
Day 1	-	-	24.0	20.0	27.0	66.0
1	4:00 am	7:00 pm	15.0	20.0	27.6	64.8
2	4:30 am	7:00 pm	14.5	20.0	26.4	67.9
3	5:30 am	7:00 pm	13.5	20.0	24.5	66.8
4	6:00 am	7:00 pm	13.0	20.0	22.1	68.5
5	7:00 am	7:00 pm	12.0	20.0	19.4	69.0
6	7:00 am	6:00 pm	11.0	10.0	18.2	75.3
7	7:00 am	5:00 pm	10.0	10.0	18.8	77.8
8	7:30 am	4:30 pm	9.0	10.0	18.4	73.1
9	7:30 am	3:30 pm	8.0	10.0	18.2	76.1
10	7:30 am	3:30 pm	8.0	10.0	18.0	75.6
11	7:30 am	3:30 pm	8.0	10.0	18.2	76.4
12	7:30 am	3:30 pm	8.0	10.0	18.2	77.4
13	7:30 am	3:30 pm	8.0	10.0	18.8	78.7
14	7:30 am	3:30 pm	8.0	10.0	18.7	78.6
15	7:30 am	3:30 pm	8.0	10.0	18.1	78.8
16	7:30 am	3:30 pm	8.0	10.0	18.3	53.1
17	7:00 am	3:30 pm	8.5	10.0	18.1	54.0
18	6:30 am	3:30 pm	9.0	10.0	17.5	63.7
19	6:30 am	4:00 pm	9.5	10.0	17.8	54.8
20	5:30 am	4:00 pm	10.5	10.0	18.3	61.2
21	4:30 am	4:00 pm	11.5	10.0	18.0	62.6
22	4:00 am	4:30 pm	12.5	10.0	17.8	62.3
23	3:30 am	5:00 pm	13.5	10.0	17.8	58.9
24 - 69	3:30 am	5:30 pm	14.0	10.0	-	-
