# Supplementary material for: Relationship between Bone Stability and Egg Production in Genetically Divergent Chicken Layer Lines
Source: Animals (Basel). 2020 May 14;10(5):850. doi: 10.3390/ani10050850 (PMC7278460; doi:10.3390/ani10050850)
Supplement: Supplementary file 1 [file animals-10-00850-s001.zip › Supplement_TableS2.pdf]

Supplementary Material

Table S2. Calculated nutrient compositions of the diets fed to the chicks during the first and second generations, respectively, and of the diet fed to the pullets during both generations.

Diet	Chicks		Pullets
Generation	1	2	1 + 2
Crude protein (%)	21.00	21.00	15.50
Crude fat (%)	4.00	4.00	4.00
Crude fibre (%)	3.20	3.50	4.50
Crude ash (%)	6.00	6.00	5.00
ME / kg DM (MJ)	11.80	11.80	11.40
Calcium (%)	1.00	0.95	0.85
Phosphorus (%)	0.70	0.65	0.55
Natrium (%)	0.15	0.15	0.15
Lysine (%)	1.20	1.20	0.65
Methionine (%)	0.48	0.48	0.34
Vitamin D3 (IU / kg)	3000.00	3000.00	1950.00
