# Supplementary material for: Relationship between Bone Stability and Egg Production in Genetically Divergent Chicken Layer Lines
Source: Animals (Basel). 2020 May 14;10(5):850. doi: 10.3390/ani10050850 (PMC7278460; doi:10.3390/ani10050850)
Supplement: Supplementary file 1 [file animals-10-00850-s001.zip › Supplement_TableS3.pdf]

Supplementary Material

Table S3. Ingredients and analysed nutrient compositions of the experimental layer diets of the first and second generations.

**Diet**	**Layers** 300 IU Vit D3		**Layers** 3000 IU Vit D3	
Generation	1	2	1	2
**Ingredients (%)**				
Wheat	39.80	39.74	39.80	39.74
Corn	20.00	20.00	20.00	20.00
Soybean, toasted	10.63	10.63	10.63	10.63
Soybean meal, toasted	8.00	8.00	8.00	8.00
High protein soybean meal, toasted	5.00	5.00	5.00	5.00
Lucerne pellets	2.44	2.44	2.44	2.44
Soybean oil	2.00	2.00	2.00	2.00
Calcium phosphate	2.16	2.46	2.16	2.46
Calcium carbonate	7.53	8.15	7.53	8.15
Sodium chloride	0.29	0.42	0.29	0.42
DL-Methionine	0.15	0.16	0.15	0.16
Silica (Sipernat®)	1.00	-	1.00	-
Premix 74237	1.00	1.00	-	-
Premix 74118	-	-	1.00	1.00
**Nutrient composition**				
Crude protein (%)	15.90	16.80	15.80	17.10
Crude fat (%)	5.60	6.20	6.20	6.20
Starch (%)	40.70	38.40	38.90	38.00
Sucrose (%)	3.60	3.00	4.00	3.40
ME / kg DM (MJ)	11.60	11.50	11.60	11.60
Vitamin D3 (IU / kg)	430.00	2000.00	2240.0	3400.00
Calcium (%)			3.38	
Phosphorus (%)			0.65	
Natrium (%)			0.26	
