# Supplementary material for: Relationship between Bone Stability and Egg Production in Genetically Divergent Chicken Layer Lines
Source: Animals (Basel). 2020 May 14;10(5):850. doi: 10.3390/ani10050850 (PMC7278460; doi:10.3390/ani10050850)
Supplement: Supplementary file 1 [file animals-10-00850-s001.zip › Supplement_TableS4.pdf]

Supplementary Material

Table S4. The effects of layer line, diet and their interaction on the observed bone traits and basic production parameters calculated within generations.

	Layer Line		Diet		Layer Line * Diet	
	F Value	p Value	F Value	p Value	F Value	p Value
**Generation 1**						
Laying maturity	169.42	<0.0001	1.20	0.2743	0.39	0.7618
Total number of eggs	346.92	<0.0001	3.39	0.0669	0.55	0.6509
Egg weight	69.00	<0.0001	0.68	0.4112	0.31	0.8152
Eggshell weight	95.23	<0.0001	0.50	0.4816	0.59	0.6203
Eggshell proportion	36.18	<0.0001	3.22	0.0740	0.30	0.8250
Total eggshell production	516.80	<0.0001	3.22	0.0739	0.33	0.8041
Daily feed consumption	76.43	<0.0001	0.24	0.6248	0.58	0.6308
Feed-to-egg conversion rate	234.46	<0.0001	0.73	0.3933	0.58	0.6271
Feed-to-eggshell conversion rate	245.59	<0.0001	2.78	0.0967	0.39	0.7589
Bone breaking strength Tibiotarsus	120.59	<0.0001	0.27	0.6054	1.60	0.1909
Bone mineral density Tibiotarsus	87.07	<0.0001	0.25	0.6155	2.86	0.0376
Weight Tibiotarsus	305.57	<0.0001	0.05	0.8319	0.33	0.8005
Length Tibiotarsus	38.36	<0.0001	0.47	0.4949	0.56	0.6442
Thickness Tibiotarsus	197.36	<0.0001	0.00	0.9924	0.28	0.8416
Bone breaking strength Humerus	55.72	<0.0001	1.66	0.1986	0.79	0.4994
Bone mineral density Humerus	43.75	<0.0001	2.50	0.1148	0.29	0.8315
Weight Humerus	63.97	<0.0001	0.03	0.8599	0.28	0.8417
Length Humerus	62.34	<0.0001	0.02	0.8808	2.02	0.1112
Thickness Humerus	40.52	<0.0001	1.03	0.3112	0.70	0.5538
Body weight 69th week of age	151.27	<0.0001	0.03	0.8624	1.52	0.2097
**Generation 2**						
Laying maturity	159.93	<0.0001	1.50	0.2211	0.10	0.9611
Total number of eggs	461.62	<0.0001	0.16	0.6909	0.51	0.6781
Egg weight	119.07	<0.0001	0.74	0.3890	0.43	0.7298
Eggshell weight	217.06	<0.0001	1.05	0.3061	1.88	0.1341
Eggshell proportion	55.17	<0.0001	0.03	0.8526	0.53	0.6613
Total eggshell production	826.22	<0.0001	1.32	0.2524	2.07	0.1049
Daily feed consumption	109.61	<0.0001	1.52	0.2181	0.99	0.3997
Feed-to-egg conversion rate	278.68	<0.0001	0.28	0.5960	3.35	0.0197
Feed-to-eggshell conversion rate	259.92	<0.0001	0.56	0.4538	2.37	0.0708
Bone breaking strength Tibiotarsus	70.35	<0.0001	0.03	0.8692	1.37	0.2536
Bone mineral density Tibiotarsus	94.81	<0.0001	0.13	0.7201	1.69	0.1706
Weight Tibiotarsus	291.45	<0.0001	0.19	0.6649	0.52	0.6672
Length Tibiotarsus	21.25	<0.0001	0.02	0.8904	0.08	0.9717
Thickness Tibiotarsus	179.68	<0.0001	0.04	0.8395	0.08	0.9711
Bone breaking strength Humerus	32.15	<0.0001	0.08	0.7807	1.30	0.2744
Bone mineral density Humerus	56.76	<0.0001	1.44	0.2315	0.19	0.9033
Weight Humerus	99.83	<0.0001	0.03	0.8528	0.21	0.8864
Length Humerus	49.52	<0.0001	0.01	0.9390	0.37	0.7765
Thickness Humerus	60.71	<0.0001	0.53	0.4689	0.61	0.6062
Body weight 69th week of age	137.20	<0.0001	1.82	0.1786	2.13	0.0963
