# Supplementary material for: Relationship between Bone Stability and Egg Production in Genetically Divergent Chicken Layer Lines
Source: Animals (Basel). 2020 May 14;10(5):850. doi: 10.3390/ani10050850 (PMC7278460; doi:10.3390/ani10050850)
Supplement: Supplementary file 1 [file animals-10-00850-s001.zip › Supplement_TableS5.pdf]

Supplementary Material

Table S5. Sample sizes for the analysis.

Variable	Total	Layer Line			
		WLA	R11	BLA	L68
Laying maturity	524	129	134	133	128
Total number of eggs	524	129	134	133	128
Egg weight	524	129	134	133	128
Eggshell weight	524	129	134	133	128
Eggshell proportion	524	129	134	133	128
Total eggshell production	524	129	134	133	128
Daily feed consumption	513	128	131	129	125
Feed-to-egg-conversion rate	513	128	131	129	125
Feed-to-eggshell conversion rate	513	128	131	129	125
Bone breaking strength Tibiotarsus	518	126	134	131	127
Bone mineral density Tibiotarsus	524	129	134	133	128
Weight Tibiotarsus	524	129	134	133	128
Length Tibiotarsus	524	129	134	133	128
Thickness Tibiotarsus	524	129	134	133	128
Bone breaking strength Humerus	516	128	131	132	125
Bone mineral density Humerus	519	129	134	128	128
Weight Humerus	521	127	134	132	128
Length Humerus	523	129	134	132	128
Thickness Humerus	523	129	134	132	128
Body weight at hatch	523	129	133	133	128
Body weight at week 21	524	129	134	133	128
Body weight at week 25	524	129	134	133	128
Body weight at week 35	524	129	134	133	128
Body weight at week 69	524	129	134	133	128
