# Supplementary material for: Relationship between Bone Stability and Egg Production in Genetically Divergent Chicken Layer Lines
Source: Animals (Basel). 2020 May 14;10(5):850. doi: 10.3390/ani10050850 (PMC7278460; doi:10.3390/ani10050850)
Supplement: Supplementary file 1 [file animals-10-00850-s001.zip › Supplement_TableS6.pdf]

Supplementary Material

Table S6. Least squares means ± standard errors and level of significance for body weight measured at hatching and different weeks of age under the effect of layer line (LL), generation (Gen) and their interaction.

	Body weight (g)				
Effect	Hatch	Week 21	Week 25	Week 35	Week 69
**Layer line (LL)**					
WLA	38.35 ± 0.37[a]	1420.02 ± 15.79[b]	1468.38 ± 16.19[b]	1497.54 ± 20.46[b]	1504.23 ± 22.26[c]
R11	33.17 ± 0.36[c]	1040.84 ± 15.60[c]	1236.40 ± 15.99[c]	1309.28 ± 20.21[c]	1362.79 ± 21.99[d]
BLA	39.35 ± 0.37[a]	1584.15 ± 15.71[a]	1663.55 ± 16.11[a]	1821.81 ± 20.34[a]	1838.10 ± 22.13[b]
L68	34.84 ± 0.37[b]	1568.91 ± 15.81[a]	1714.92 ± 16.21[a]	1837.91 ± 20.48[a]	1923.44 ± 22.29[a]
**Generation (Gen)**					
Gen 1	35.86 ± 0.26	1379.01 ± 11.17	1485.67 ± 11.45	1567.49 ± 14.47	1616.21 ± 15.76
Gen 2	37.00 ± 0.26	1427.95 ± 11.07	1555.96 ± 11.36	1665.79 ± 14.34	1698.07 ± 15.59
**LL x Gen**					
WLA x Gen1	37.77 ± 0.52	1376.72 ± 22.36	1415.84 ± 22.91	1460.33 ± 28.96	1443.48 ± 31.51
WLA x Gen2	38.93 ± 0.52	1463.31 ± 22.32	1520.93 ± 22.88	1534.75 ± 28.91	1564.98 ± 31.45
R11 x Gen1	32.64 ± 0.51	1027.33 ± 21.99	1222.77 ± 22.53	1284.20 ± 28.49	1338.12 ± 31.02
R11 x Gen2	33.69 ± 0.52	1054.36 ± 22.14	1250.04 ± 22.71	1334.37 ± 28.66	1387.45 ± 31.17
BLA x Gen1	38.84 ± 0.52	1549.66 ± 22.41	1627.91 ± 22.97	1767.90 ± 29.03	1804.33 ± 31.60
BLA x Gen2	39.87 ± 0.51	1618.63 ± 22.02	1699.19 ± 22.59	1875.73 ± 28.50	1871.86 ± 30.98
L68 x Gen1	34.18 ± 0.52	1562.32 ± 22.61	1676.16 ± 23.16	1757.53 ± 29.31	1878.89 ± 31.92
L68 x Gen2	35.51 ± 0.51	1575.50 ± 22.11	1753.67 ± 22.68	1918.30 ± 28.62	1968.00 ± 31.12

ANOVA significance level (p value)

	Layer line	Generation	LL x Gen
Hatch	<0.0001	0.0019	0.9908
Week 21	<0.0001	0.0020	0.3097
Week 25	<0.0001	<0.0001	0.3907
Week 35	<0.0001	<0.0001	0.2486
Week 69	0.0003	<0.0001	0.6892

Means within a column with different letters differ significantly (Tukey's HSD-Test, $p < 0.05$).
